# Regulation of Sphingolipid Biosynthesis by the Morphogenesis Checkpoint Kinase Swe1*

**DOI:** 10.1074/jbc.M115.693200

**Published:** 2015-12-03

**Authors:** Neha Chauhan, Gongshe Han, Niranjanakumari Somashekarappa, Kenneth Gable, Teresa Dunn, Sepp D. Kohlwein

**Affiliations:** From the ‡ Institute of Molecular Biosciences, BioTechMed-Graz, University of Graz, Humboldtstrasse 50/II, 8010 Graz, Austria and; the § Department of Biochemistry, Uniformed Services University of the Health Sciences, Bethesda, Maryland 20814

**Keywords:** cell cycle, metabolic regulation, phosphorylation, serine palmitoyltransferase, sphingolipid, yeast

## Abstract

Sphingolipid (SL) biosynthesis is negatively regulated by the highly conserved endoplasmic reticulum-localized Orm family proteins. Defective SL synthesis in *Saccharomyces cerevisiae* leads to increased phosphorylation and inhibition of Orm proteins by the kinase Ypk1. Here we present evidence that the yeast morphogenesis checkpoint kinase, Swe1, regulates SL biosynthesis independent of the Ypk1 pathway. Deletion of the Swe1 kinase renders mutant cells sensitive to serine palmitoyltransferase inhibition due to impaired sphingoid long-chain base synthesis. Based on these data and previous results, we suggest that Swe1 kinase perceives alterations in SL homeostasis, activates SL synthesis, and may thus represent the missing regulatory link that controls the SL rheostat during the cell cycle.

## Introduction

As cells grow and divide, a strict balance of a large variety of lipid species needs to be established and maintained to meet the metabolic demands to support growth in response to changes in environmental conditions. Sphingolipids (SLs)^4^ along with sterols and glycerophospholipids are integral constituents of the lipid phase of membrane bilayers in all eukaryotic cells. In addition, SLs play key roles in a plethora of cellular processes (1–5), including nutrient uptake (6), cellular trafficking and stress signaling (7), calcium signaling (8), and aging (9). In mammals, imbalanced SL homeostasis is associated with several metabolic disorders like diabetes, cardiovascular and respiratory diseases, inflammation, Alzheimer disease, and cancer (10–18).

Numerous studies also suggest the involvement of sphingolipids in the regulation of the cell cycle (19–25). In mammals, ceramides were shown to regulate both the G1 (Gap-1) and G_2_/M (Gap-2/mitotic) progression of the cell cycle (26–28), and inhibition of SLs is a potential therapeutic target for tumor suppression by induction of a G_2_/M cell cycle arrest (26). In yeast, overexpression of dihydrosphingosine-1-phosphate phosphatase encoded by *YSR2/3* leads to a G_1_ cell cycle arrest (24). Reduced SL levels affect the integrity of the actin cytoskeleton and establishment of cell polarity (29, 30), and absence of LCBs blocks the initiation of bud formation, may lead to a G_2_ cell cycle arrest, and also blocks cytokinesis (31–34). Notably, sphingolipid metabolism is also frequently implicated in the cellular response to stress. In order to overcome heat stress, a transient G_0_/G_1_ cell arrest and increased synthesis of LCBs are required. Upon stress, cells accumulate large amounts of phytosphingosine (PHS), in particular C20-PHS (23, 35, 36), which was also found to accumulate in cells entering the stationary phase (37).

**FIGURE 1.**
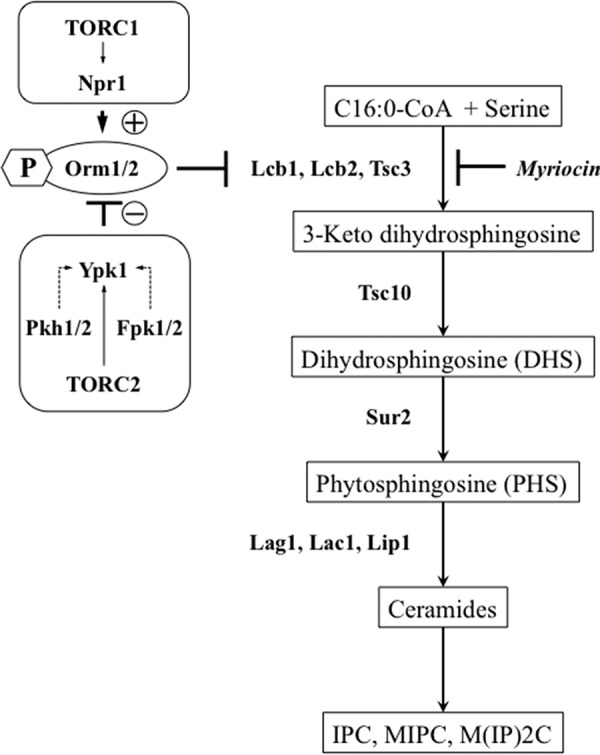
**Schematic pathways of yeast sphingolipid biosynthesis.** SPT (Lcb1, Lcb2, and Tsc3) is potently inhibited by the inhibitor myriocin. Orm proteins negatively regulate SPT and influence SL levels. The Orm proteins, in turn, are regulated by kinases Ypk1 and Npr1, which are under control of the TORC2 and TORC1 kinase pathways, respectively.

Based on the observations that both SL overproduction and deficiency may cause defective cell growth and cell cycle progression, the picture emerged of a sphingolipid rheostat that tightly regulates the amount of (bioactive) SLs (1). The yeast *Saccharomyces cerevisiae* has been instrumental in identifying key components that drive cell cycle progression and also the regulatory mechanisms involved in SL metabolism, which are both processes that are highly conserved in eukaryotes. SL synthesis is initiated by serine palmitoyltransferase (SPT), which is encoded by *LCB1/LCB2* (catalytic subunit) and *TSC3* (regulatory subunit) and which catalyzes the condensation of serine and palmitoyl-CoA to LCBs; these are further metabolized to ceramides and complex SLs (38–41). SPT is part of the multimeric SPOTS complex composed of Lcb1, Lcb2, Tsc3, Sac1, and the regulatory proteins Orm1/2 (42, 43). The yeast Orm1 and Orm2 proteins belong to the highly conserved *ORMDL* gene family, which encompasses three homologs in humans, namely *ORMDL1/2/3* (44). Single nucleotide polymorphisms at chromosome 17q21 near *ORMDL3* are associated with increased risk of childhood asthma in multiple ethnic groups (45–48). In yeast, multisite phosphorylation of the Orm proteins alleviates their repressing effect on SPT and provides a finely tunable mechanism for the regulation of SL synthesis. However, recent evidence suggests that Orm proteins may have additional functions in sphingolipid metabolism beyond regulating SPT activity (49). Phosphorylation of Orm proteins in yeast is regulated via the target of rapamycin (TOR) pathways, which interconnects SL metabolism with major regulatory networks. The TORC1 and the TORC2 pathways, however, function independently of each other in their regulatory impact on Orm proteins (49, 50). SL depletion activates TORC2 and its downstream kinase Ypk1, the functional ortholog of mammalian serum/glucocorticoid-regulated kinase SGK. Orm1/2 phosphorylation by Ypk1 prevents inactivation of SPT and therefore increases SL synthesis (50). Inhibition of the nutrient-sensitive TORC1 pathway, on the other hand, activates its downstream kinase Npr1, which in turn phosphorylates and inactivates Orm proteins, promoting the synthesis of complex SLs in the Golgi (49) (Fig. 1).

Large scale studies have indicated a possible biochemical interaction between the Orm proteins and Swe1 kinase (51), and evidence suggests that combined defects in Lcb1 and Swe1 kinase function are detrimental to the cell (52, 53). Swe1 kinase is an important cell cycle checkpoint in yeast, indicating a possible connection between cell cycle progression and the regulation of sphingolipid metabolism. Swe1 either arrests or delays the cell cycle when essential events in cell cycle progression are defective (54, 55) by phosphorylating and inhibiting the major cyclin-dependent kinase, Cdc28 (56–59). Swe1 kinase functions as a checkpoint by assessing cell polarity (60), defects in actin cytoskeleton (61), bud emergence (62), bud size (63), cell size and membrane growth (64), septin defects (65), and DNA replication stress (66). It also regulates anaphase onset (67), pachytene checkpoint (68), Hsp90 phosphorylation (69), and spindle pole body separation (70). Numerous other functions have been assigned to Swe1 kinase to mediate survival under high osmolarity conditions (71), proper inheritance of cortical ER (72), calcineurin-mediated cell cycle control (73), cardiolipin synthesis (74), and surviving genotoxic stress by responding to changes in levels of complex SLs (54). Thus, it is apparent that Swe1 kinase, although not essential for cell survival, plays regulatory roles in numerous cell cycle-related processes. Indeed, in a recent study, we have shown that Swe1 phosphorylates and inhibits Cdc28 in response to defective lipolysis, which is a source of lipid precursors for sphingolipid synthesis (75, 76). Here we show that the Swe1 checkpoint kinase positively regulates SPT, presumably by phosphorylating Orm2, independently of Ypk1. Because Swe1 activity is regulated by SLs, we propose an autoregulatory loop that regulates cell cycle progression in response to alterations in SL metabolism.

## Experimental Procedures

### 

#### 

##### Strains

The *S. cerevisiae* strains used in this study are listed in Table 1. Double mutants were constructed by standard genetic crosses and tetrad dissection; the triple mutants were generated by replacing the designated ORFs with resistance cassettes by homologous recombination. Gene deletions were verified by colony PCR with the appropriate up-tag and down-tag primers. The *cdc28*^Y19F^ allele was introduced into the BY strain background in two steps. First the mutant CDC28 ORF was PCR-amplified from genomic DNA prepared from the *MAT**a** cdc28^Y19F^ TRP1 GAL-SWE1myc*::*URA3* strain (kind gift from the laboratory of Daniel Lew) using primers 5′-GACTAATGCATCATGGCTTATGTATTATACTTGCTTATGT-3′and 5′-ACTAATGCATGCTCCTAACGGTTGGTCCTTTGGAATACC-3′. The amplified fragment was cut with NsiI and inserted into the pSH47/NAT^R^ plasmid. This plasmid was used as a template for PCR amplification of the *cdc28^Y19F^* ORF along with the neighboring NatR selection marker using primers 5′-TTTTTATACAATACATATATATATATATATATATATATTTACAAGAAAAGACATGGAGGCCCAGAATACCC-3′ and 5′-GCTCCTAACGGTTGGTCCTTTGGAATACC-3′. The PCR product was chromosomally integrated into wild type, *swe1*, *ypk1*, and *ypk2* strains, and transformants were selected for ClonNAT resistance, as described previously (75). The mutation was confirmed by sequencing.

**TABLE 1:** **Strains used in this study**

Strain	Genotype	Source
BY4742	*MAT*α *his3*Δ*1 leu2*Δ*0 lys2*Δ*0 ura3*Δ*0*	Euroscarf
YNC001	*MAT*α *his3*Δ*1 leu2*Δ*0 lys2*Δ*0 ura3*Δ*0 swe1*::*KanMX4*	Open Biosystems
YNC003	*MAT*α *his3*Δ*1 leu2*Δ*0 lys2*Δ*0 ura3*Δ*0 cdc28^Y19F^*::*Nat^R^*	Ref. 74
YNC012	*MAT*α *his3*Δ*1 leu2*Δ*0 lys2*Δ*0 ura3*Δ*0 ypk1*::*KanMX4*	Euroscarf
YNC013	*MAT*α *his3*Δ*1 leu2*Δ*0 lys2*Δ*0 ura3*Δ*0 ypk2*::*KanMX4*	Euroscarf
YNC014	*MAT*α *his3*Δ*1 leu2*Δ*0 lys2*Δ*0 ura3*Δ*0 ypk1*::*KanMX4 swe1*::*HIS3*	This study
YNC015	*MAT*α *his3*Δ*1 leu2*Δ*0 lys2*Δ*0 ura3*Δ*0 ypk2*::*KanMX4 swe1*::*HIS3*	This study
YNC016	*MAT****a*** *his3*Δ*1 leu2*Δ*0 lys2*Δ*0 ura3*Δ*0 orm1*::*KanMX4 orm2*::*KanMX4*	This study
YNC017	*MAT****a*** *his3*Δ*1 leu2*Δ*0 lys2*Δ*0 ura3*Δ*0 swe1*::*Nat^R^ orm1*::*KanMX4 orm2*::*KanMX4*	This study
YNC018	*MAT****a*** *his3*Δ*1 leu2*Δ*0 lys2*Δ*0 ura3*Δ*0 orm1*::*KanMX4 orm2*::*KanMX4 cdc28 ^Y19F^*::*Nat^R^*	This study
DDY5142	*MAT*α *his3-*Δ*200 leu2-3,112 ura3-52 ypk1*Δ::*CgLEU2 ypk2*Δ::*CgHIS3 ura3-52*::*ypk1-as*::*URA3 FLAG-ORM2*::*Nat^R^*	Ref. 34
YNC019	*MAT*α *his3-*Δ*200 leu2-3,112 ura3-52 ypk1*Δ::*CgLEU2 ypk2*Δ::*CgHIS3 ura3-52*::*ypk1-as*::*URA3 swe1*:: *KanMX4 FLAG-ORM2*::*Nat^R^*	This study
YNC020	*MAT*α *his3*Δ*1 leu2*Δ*0 lys2*Δ*0 ura3*Δ*0* (transformed with pYEX4T-1)	This study
YNC021	*MAT*α *his3*Δ*1 leu2*Δ*0 lys2*Δ*0 ura3*Δ*0 swe1*::*KanMX4* (transformed with pYEX4T-1)	This study
YNC022	*MAT*α *his3*Δ*1 leu2*Δ*0 lys2*Δ*0 ura3*Δ*0 swe1*::*KanMX4* (transformed with pYEX4T-1-*SWE1*)	This study
YNC023	*MAT*α *his3*Δ*1 leu2*Δ*0 lys2*Δ*0 ura3*Δ*0 swe1*::*KanMX4* (transformed with pYEX4T-1-*YPK1*)	This study
YNC024	*MAT*α *his3*Δ*1 leu2*Δ*0 lys2*Δ*0 ura3*Δ*0 cdc28^Y19F^*::*Nat^R^* (transformed with pYEX4T-1)	This study
YNC025	*MAT*α *his3*Δ*1 leu2*Δ*0 lys2*Δ*0 ura3*Δ*0 cdc28^Y19F^*::*Nat^R^* (transformed with pYEX4T-1-*SWE1)*	This study
YNC026	*MAT*α *his3*Δ*1 leu2*Δ*0 lys2*Δ*0 ura3*Δ*0 cdc28^Y19F^*::*Nat^R^* (transformed with pYEX4T-1*-YPK1*)	This study
YNC027	*MAT*α *his3*Δ*1 leu2*Δ*0 lys2*Δ*0 ura3*Δ*0 ypk1*::*KanMX4* (transformed with pYEX4T-1)	This study
YNC028	*MAT*α *his3*Δ*1 leu2*Δ*0 lys2*Δ*0 ura3*Δ*0 ypk1*::*KanMX4* (transformed with pYEX4T-1-*SWE1*)	This study
YNC029	*MAT*α *his3*Δ*1 leu2*Δ*0 lys2*Δ*0 ura3*Δ*0 ypk1*::*KanMX4* (transformed with pYEX4T-1-*YPK1*)	This study
YNC030	*MAT*α *his3*Δ*1 leu2*Δ*0 lys2*Δ*0 ura3*Δ*0 ypk1*::*KanMX4 cdc28^Y19F^*::*Nat^R^* (transformed with pYEX4T-1)	This study
YNC031	*MAT*α *his3*Δ*1 leu2*Δ*0 lys2*Δ*0 ura3*Δ*0 ypk1*::*KanMX4 cdc28^Y19F^*::*Nat^R^* (transformed with pYEX4T-1-*SWE1*)	This study
TDY4071	*MAT*α *his3*Δ*1 leu2*Δ*0 lys2*Δ*0 ura3*Δ*0 tsc3*::*NatR*	Ref. 90
YNC032	*MAT*α *his3*Δ*1 leu2*Δ*0 lys2*Δ*0 ura3*Δ*0 tsc3*::*NatR swe1*::*URA3*	This study

For constructing an *N*-terminal fusion with glutathione *S*-transferase (GST), the *SWE1* gene was amplified as a BamHI-NotI fragment by PCR using genomic DNA as the template and primers 5′-GACTAGGATCCAGTTCTTTGGACGAGGATGAAGAG-3′ and 5′-GACTAGCGGCCGCTCATATAAAAAATTTTGGCTTAGGTCCAAA-3′. The PCR fragment was inserted into the respective restriction sites of plasmid pYEX4T-1 (Clontech) downstream of the copper-inducible *CUP1* promoter. The GST-Ypk1 fusion under control of the *CUP1* promoter was constructed by the same strategy using primers 5′-GACTAGGATCCTATTCTTGGAAGTCAAAGTTTAAGTTTG-3′ and 5′-GACTAGCGGCCGCCTATCTAATGCTTCTACCTTGCACC-3′.

For the generation of 3xFLAG-Orm2, a fragment of *ORM2* (191 bp upstream and 189 bp downstream of *ORM2* ORF, with N-terminal BamHI and C-terminal XhoI sites) was amplified from yeast genomic DNA and ligated into the pRS316 vector. An NheI restriction site was introduced after the start codon of *ORM2* by QuikChange mutagenesis (Agilent Technologies). A fragment of NheI/XhoI-flanked 3xFLAG-*ORM2* obtained by PCR was used to replace the untagged *ORM2* with an N-terminal 3xFLAG-tagged *ORM2* (under the *ORM2* promoter) by homologous recombination.

##### Media and Growth Conditions

Yeast cells were grown in YPD medium containing 1% yeast extract, 2% peptone, and 2% glucose or in YNB minimal medium containing 0.67% yeast nitrogen base, 2% glucose, and the indicated amino acids (77). Transformed yeast strains carrying expression plasmids were maintained on uracil drop-out medium. YPD plates containing 200 mg/liter G418 (Calbiochem) or 100 mg/liter nourseothricin (Sigma) were used to select for Geneticin or ClonNAT resistance, respectively. Yeast strains generated by introducing the *HIS3* gene at the designated ORF were selected on histidine drop-out medium. Solid media had the same composition plus 2% agar. Sporulation medium contained 0.25% yeast extract, 0.1% glucose, and 1% potassium acetate. To inhibit *de novo* synthesis of SLs (78), YNB minimal media were supplemented with myriocin (Sigma), added from DMSO stock solutions, at the indicated concentrations. A stock of phytosphingosine (C18-PHS from yeast; Sigma-Aldrich) was prepared in methanol and supplemented to minimal media containing 0.1% Tergitol (Sigma) at a final concentration of 15 μm.

##### Myriocin Sensitivity Tests

Sublethal concentrations of the SPT inhibitor myriocin (20–50, 100–250, and 300–500 ng/ml) were used to analyze the sensitivity of different yeast strains toward SPT inhibition. Yeast strains were cultured overnight at 30 °C in YNB minimal medium or YNB uracil drop-out medium, and 10 *A*_600_ units of cells were harvested and then serially diluted 1:10 in water. 5 μl of each serial dilution were dropped onto myriocin- or myriocin + phytosphingosine (PHS)-containing agar plates. The same amount of DMSO was added to the control plates. Growth was monitored after 3–4 days of incubation at 30 °C.

##### Analysis of in Vivo Phosphorylation of Orm2

Yeast strains *ypk1^as^ ypk2* FLAG-Orm2 and *ypk1^as^ ypk2 swe1* FLAG-Orm2 were precultured for 72 h, harvested by centrifugation, and then released into fresh minimal media containing 670 ng/ml myriocin to induce phosphorylation of Orm2. After 30 min of myriocin treatment (0 min time point), cells were then additionally treated with 50 μm 3MB-PP1 (Calbiochem) Ypk1^as^ kinase inhibitor for 30 min. Three *A*_600_ units of cells were harvested at the indicated time points by centrifugation at 4,000 rpm for 5 min at 4 °C. The cell pellets were resuspended in ice-cold 12.5% TCA and frozen at −80 °C overnight. The samples were thawed on ice, and the TCA was removed by centrifugation. The pellet was washed twice with ice-cold 80% acetone, air-dried, and resuspended in Laemmli solution (0.1 m NaCl, 1% SDS) and loading dye and boiled for 5 min (79). The proteins were resolved on a 15% Phos-tag gel containing 25 μm Phos-tag (80) and 50 μm MnCl_2_. The Phos-tag gel was washed twice for 10 min with transfer buffer containing 2 mm EDTA, followed by a 10-min wash with transfer buffer without EDTA. The proteins were transferred onto a nitrocellulose membrane (Bio-Rad) and probed with anti-FLAG M2 peroxidase (HRP-conjugated) antibody (Sigma) and detected using ECL Western blotting substrate (Pierce).

##### Preparation of Yeast Whole Cell Extracts and Western Blotting of GST-Swe1

0.15 *A*_600_ units of the mutant strains *swe1* and *cdc28^Y19F^* overexpressing GST-*SWE1* were inoculated in minimal medium (−Ura) and cultivated to the logarithmic growth phase. Expression of GST-*SWE1* was induced for 2 h by the addition of 50 μm copper sulfate. Cells were treated with myriocin (670 ng/ml) or PHS (15 μm) for 60 min. Three *A*_600_ units of cells were harvested, and proteins were precipitated as described above. Proteins were resolved on 8% SDS-polyacrylamide gels and blotted onto nitrocellulose membranes (Bio-Rad). Anti-GST antibody (GE Healthcare) was used to detect GST-Swe1. Horseradish peroxidase-conjugated anti-goat IgG (Chemicon) was the secondary antibody, and ECL Western blotting Substrate (Pierce^TM^) was used for detection. Coomassie staining of the gel served as loading control.

##### Extraction of LCBs from Yeast Cells

15 *A*_600_ units of cells were harvested from a logarithmically growing culture by centrifugation and washed with water. The pellet was resuspended in 2 m NH_4_OH and 2 ml of chloroform/methanol (1:2); C17-dihydrosphingosine internal standard was added (Sigma). Cells were lysed by vortexing at high speed for 3–4 min in the presence of zirconium beads. The supernatant was collected into fresh Pyrex tubes, and 1.5 ml of chloroform and 3 ml of 0.5 m NH_4_OH were added. After vortexing and a 5-min centrifugation at 4,000 rpm, the top layer was aspirated, and to the bottom layer another 1 ml of chloroform and 3 ml of 60 mm KCl were added. The top layer was again aspirated after centrifugation; the bottom layer was dried under a stream of nitrogen and resuspended in 100 μl of methanol, 190 mm triethylamine (2:0.3, v/v). 80 μl of extract were transferred to an HPLC vial containing 20 μl of AccQ reagent (Waters) and allowed to react for 30 min at room temperature. Aminophospholipids were deacylated by adding 5 μl of 1 m KOH in methanol and incubation at 37 °C for 30 min. The samples were neutralized by the addition of 5 μl of 1 m acetic acid in methanol. Insoluble materials were removed by brief centrifugation, and the supernatant was transferred to a new HPLC vial. Analysis was performed using an HP Series II 1090 liquid chromatograph with HP Chemstation coupled to an Agilent 1100 series fluorescence detector, as described (81).

##### Microscopy

Logarithmically growing cells were harvested and imaged with a Leica SP2 confocal microscope (Leica Microsystems, Inc.) using a 100× numerical aperture 1.4 oil objective and differential interference contrast optics.

## Results

### 

#### 

##### The Morphogenesis Checkpoint Kinase Swe1 Regulates Serine Palmitoyltransferase Activity

**FIGURE 2.**
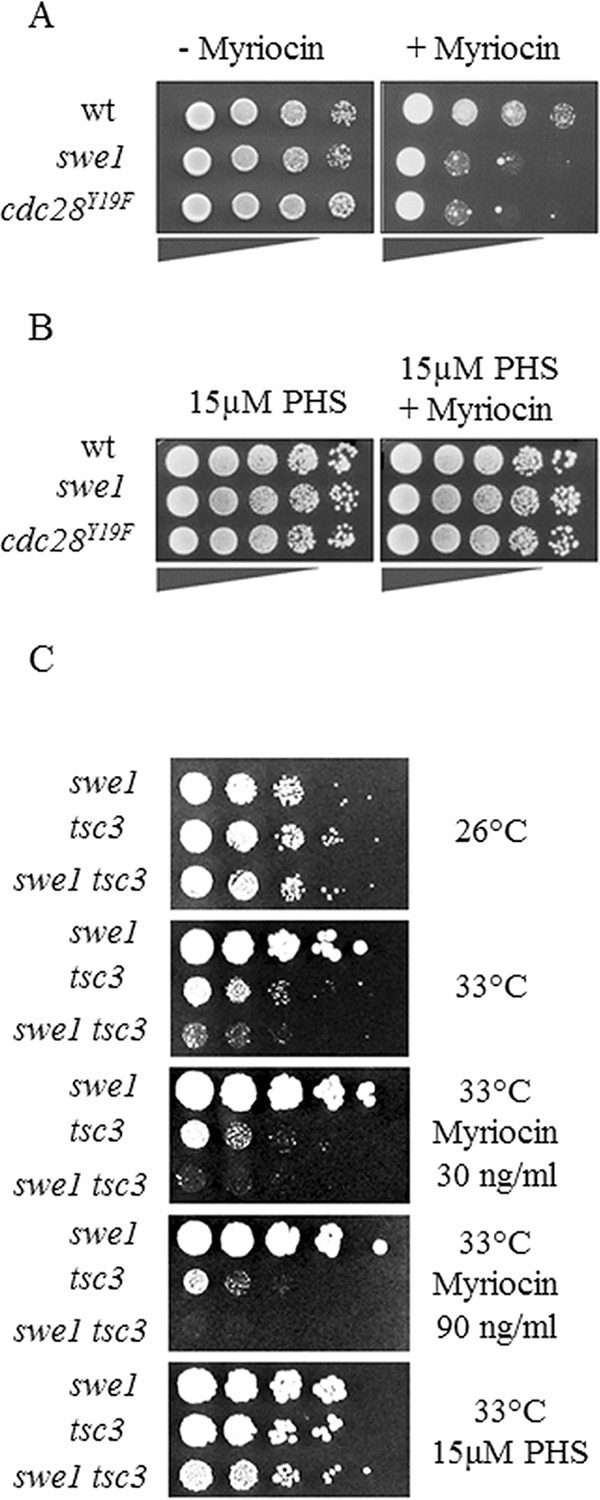
**Inactivation of the Swe1 kinase cell cycle checkpoint renders cells sensitive to myriocin.** *A*, wild type, *swe1*, and *cdc28^Y19F^* mutants were analyzed for growth on minimal medium supplemented with 100–250 ng/ml myriocin in DMSO. The control plate contained DMSO only. *B*, wild type, *swe1*, and *cdc28^Y19F^* mutants were analyzed for growth in the presence of 250 ng/ml myriocin and 15 μm PHS in 0.1% Tergitol. The control plate contained 15 μm PHS in 0.1% Tergitol. *C*, *swe1*, *tsc3*, and *swe1 tsc3* double mutants were analyzed for growth at 26 and 33 °C and in the presence of increasing concentrations of myriocin. Representative data of biological triplicates are shown.

Recent high-throughput studies indicated that *swe1* and *lcb1^ts^* mutants show a negative genetic interaction, suggesting that the cell cycle checkpoint responds to alterations in sphingolipid metabolism (52, 53). If this was the case, then specific inhibition of SPT by myriocin (73) would be expected to phenocopy this growth defect in a mutant strain lacking *SWE1*. The *swe1* deletion mutant indeed showed a severe growth defect in the presence of sublethal doses of myriocin as compared with the wild type (Fig. 2*A*). Thus, the absence of the morphogenesis checkpoint kinase Swe1 renders cells highly sensitive to reduced levels of sphingolipids. Swe1 asserts its checkpoint regulation by phosphorylating and inhibiting cyclin-dependent kinase Cdc28 at its tyrosine 19 residue; upon inhibition of Cdc28, the cell cycle is arrested/delayed until all of the conducive conditions are met (56, 57, 59). To test whether the observed growth defect of *swe1* mutants in the presence of myriocin was due to lack of Cdc28^Tyr-19^ phosphorylation, we made use of a *cdc28^Y19F^* mutant that escapes this regulation by Swe1; in this mutant, the morphogenesis checkpoint cannot be activated. The *cdc28^Y19F^* mutant, like the *swe1* mutant, showed a severe growth defect in the presence of myriocin (Fig. 2*A*), further supporting the notion that the cell cycle checkpoint activation is linked to the abundance of SLs. Conversely, these results suggest that the absence of the Swe1 kinase checkpoint or lack of phosphorylation of its downstream target Cdc28 leads to decreased levels of SLs and loss of viability upon SPT inhibition. Thus, Swe1-dependent phosphorylation/inhibition of Cdc28 appears to be required to attenuate cell cycle progression until a certain SL threshold level is achieved. There is no evidence, however, that Ccd28 directly regulates SL synthesis.

Swe1 is an important cell cycle checkpoint, so we were concerned that the myriocin-induced growth defect observed in the *swe1* mutant could be a pleiotropic effect unlinked to SL metabolism. To exclude this possibility, we performed the growth tests on medium plates co-supplemented with myriocin and the sphingoid long-chain base, PHS, that bypasses the SPT reaction. Indeed, PHS supplementation rescued the *swe1* and *cdc28^Y19F^* growth defect in the presence of myriocin, indicating that the levels of LCBs are growth-limiting in the mutant strains (Fig. 2*B*). Further support for this assumption comes from growth experiments of strains lacking the activating SPT subunit, Tsc3 (39): *tsc3* mutants are sensitive for growth at 33 °C, but growth of the *swe1 tsc3* double mutants was virtually abolished. Rescue of the growth defect by PHS supplementation that bypasses the SPT defect further supports the notion that Swe1 regulates SL homeostasis (Fig. 2*C*).

##### Swe1 Kinase Overexpression Rescues the Growth Defect Caused by the Inhibition of SPT

**FIGURE 3.**
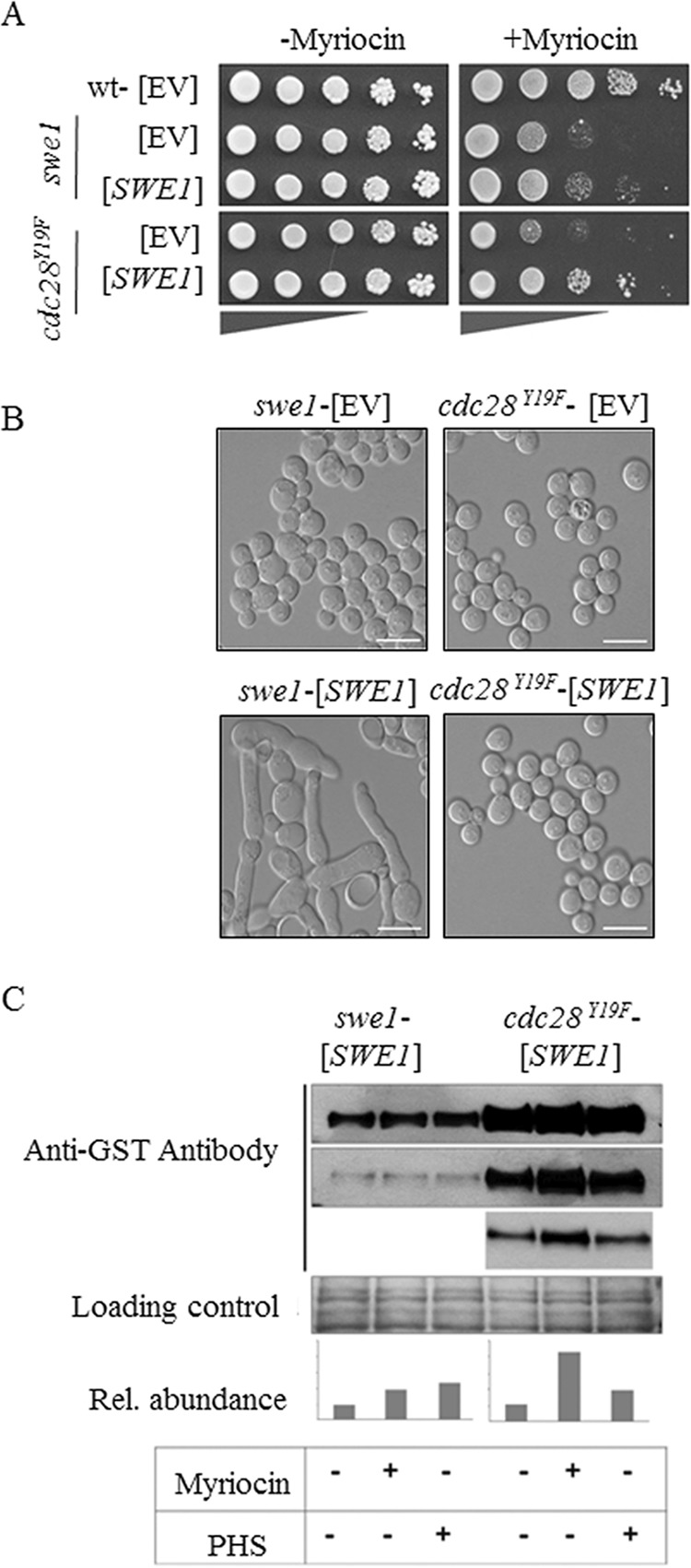
**The overexpression of *SWE1* rescues the myriocin-induced growth defect.** *A*, *SWE1* was overexpressed in the *swe1* (*swe1*-[*SWE1*]) and *cdc28^Y19F^* (*cdc28^Y19F^*-[*SWE1*]) mutants under the control of the *CUP1* promoter and analyzed for growth on media supplemented with 100–250 ng/ml myriocin. Control strains contained the pYEX4T-1 empty vector (*EV*). *B*, the morphologies of *swe1* and *cdc28^Y19F^* mutant strains overexpressing either the empty vector (-[*EV*]) or *SWE1* (-[*SWE1*]) were analyzed by microscopy. *Scale bar* = 10 μm. *C*, determination of GST-Swe1 levels by Western blotting. The *bottom panels* show the same blot exposed for a shorter period of time. A Coomassie-stained SDS-polyacrylamide gel is shown as a loading control; bar graphs indicate the relative abundance of signals from the same blot.

We next tested whether overexpression of the Swe1 kinase rendered cells resistant to myriocin, which would indicate a direct role of the kinase in regulating SL synthesis. Because overexpression of Swe1 in wild type cells induces a cell cycle arrest due to increased phosphorylation of Cdc28 (82), we also tested Swe1 overexpression in *cdc28^Y19F^* mutant cells, which escape regulation by Swe1. As shown in Fig. 3*A*, Swe1 overexpression in the *swe1* mutant cells led to only a minor increase in myriocin resistance, presumably due to the counterbalancing effects of increasing myriocin resistance by derepressing SPT while at the same time activating the cell cycle checkpoint by Cdc28 phosphorylation. Indeed, overexpression of Swe1 in the *cdc28^Y19F^* mutant restored myriocin resistance back to wild type levels (Fig. 3*A*), indicating an activation of cellular SL production. Moreover, overexpression of *SWE1* in wild-type cells results in an elongated cell phenotype (Fig. 3*B*) and an extended cell cycle delay/arrest due to prolonged inhibition of Cdc28 (58, 67, 82). This morphology defect is not observed upon overexpression of *SWE1* in the *cdc28^Y19F^* mutant (Fig. 3*B*). Interestingly, the level of GST-tagged Swe1 protein was much lower when expressed from a plasmid in *swe1* mutants as compared with the *cdc28^Y19F^* mutant, confirming previous studies that showed a feedback regulation of Swe1 abundance by Cdc28 activity (56). On the other hand, Swe1 protein levels were significantly increased in the presence of myriocin in both mutant strains (Fig. 3*C*), suggesting a regulation of Swe1 expression/abundance by the levels of SLs. In addition, PHS supplementation attenuated GST-Swe1 abundance in the *cdc28^Y19F^* mutant, further supporting previous observations that Swe1 responds to altered sphingolipid metabolism (75).

##### Sphingoid Long-chain Base Levels Are Reduced in Mutants Lacking Swe1 Kinase

**FIGURE 4.**
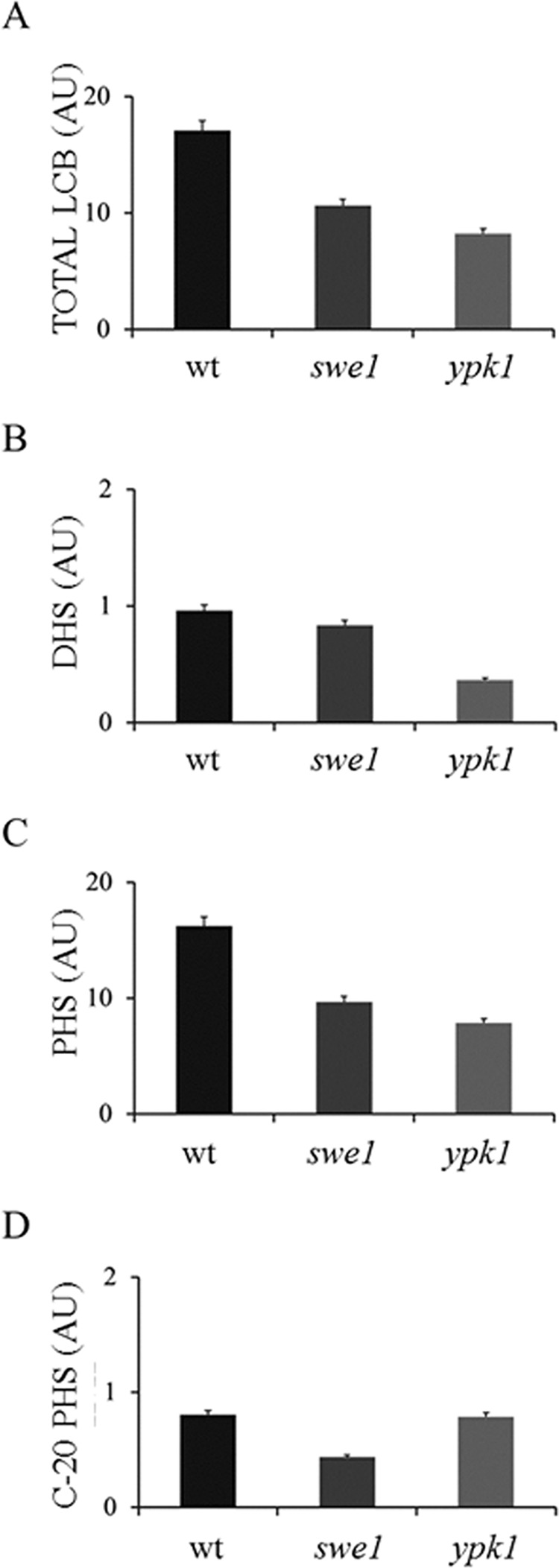
**Levels of LCBs are reduced upon deletion of the Swe1.** *A*, total LCB levels in *swe1* and *ypk1* mutants as compared with the wild type. *B*, relative distribution of dihydrosphingosine in wild type, *ypk1*, and *swe1* mutants. *C*, relative distribution of PHS in wild type, *ypk1*, and *swe1* mutants. *D*, relative distribution of C20-PHS in wild type, *ypk1*, and *swe1* mutants. *Error bars*, S.D. of three independent experiments. *AU*, arbitrary units.

To better understand the correlation between Swe1 activity and SL metabolism, we next analyzed the levels of total LCBs in the *swe1* deletion mutant. Mutants lacking a known regulator of SPT activity, Ypk1 (see below) were used as a positive control. Indeed, the total levels of LCBs were significantly lower in the *swe1* mutant compared with wild type and similar to the *ypk1* deletion mutant (Fig. 4*A*) (50, 83). Dihydrosphingosine content was comparable in wild type and the *swe1* mutant, whereas the *ypk1* mutant showed a significant reduction in dihydrosphingosine levels (Fig. 4*B*); PHS levels were significantly reduced in both *swe1* and *ypk1* mutants as compared with wild type (Fig. 4*C*). A closer analysis of the PHS species revealed that deletion of *SWE1* drastically reduced the levels of C20-PHS in comparison with both the wild type and the *ypk1* mutant (Fig. 4*D*). Previous studies showed that C20-PHS levels strongly respond to exposure of cells to heat stress (84, 85). The SL composition of both *swe1* and *ypk1* mutants indicates a similar, but not identical, physiological impact of the two kinases on reducing total long-chain base levels.

##### In Vivo Phosphorylation of Orm2 by Swe1

**FIGURE 5.**
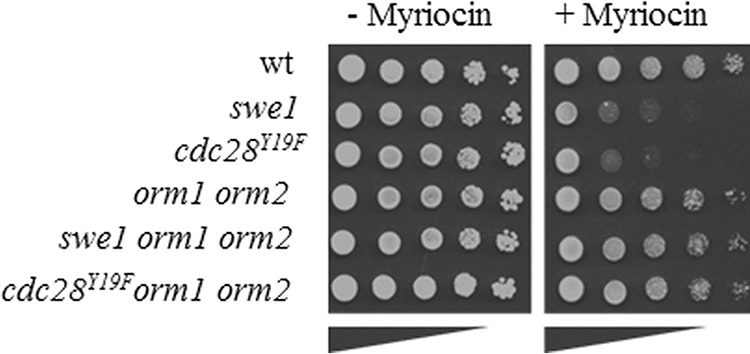
**The growth defect of *swe1* mutants in the presence of myriocin is due to reduced SPT activity.** Wild type, *swe1*, *orm1 orm2, swe1 orm1 orm2*, and *cdc28^Y19F^ orm1 orm2* mutants were analyzed for growth in the presence of 100–250 ng/ml myriocin. Representative data from three independent experiments are shown.

Because Swe1 overexpression rescued the myriocin-induced growth defect in the *cdc28^Y19F^* mutant, we assumed that Swe1 has another target besides Cdc28 that directly or indirectly regulates the synthesis of SLs. The first and committed step in SL synthesis is catalyzed by SPT, whose activity is negatively regulated by Orm1 and Orm2 (42, 43) and the activating regulatory subunit, Tsc3 (39). To test whether Swe1 and the Orm proteins functionally interact, we generated *swe1 orm1 orm2* and *cdc28^Y19F^ orm1 orm2* triple mutants and tested their sensitivity to myriocin. As shown in Fig. 5, additional deletion of the Orm proteins in the *swe1* and *cdc28^Y19F^* mutant backgrounds restored growth in the presence of myriocin, linking Swe1 kinase function directly to the regulation of SPT activity.

Orm proteins are heavily phosphorylated by Ypk1 and, to some extent, also by Ypk2, which attenuates their inhibitory activity on SPT (30), and lack of Ypk1 alone or both Ypk1 and Ypk2 results in increased myriocin sensitivity or loss of viability, respectively. Both phenotypes are rescued by the additional deletion of *ORM2* (deletion of *ORM1* alone weakly suppresses growth of the *ypk1ypk2* double mutant) or by supplementation of cells with PHS. Thus, Ypk1 (and Ypk2) activity is essential to phosphorylate and inactivate the Orm1/2 proteins to sustain SPT activity. Based on the growth tests in the presence of myriocin and the suppressive effects of deleting both *ORM* genes, we speculated that Swe1 may also play a role in phosphorylating and inhibiting the Orm proteins, similar to the Ypk1 kinase. According to this model, lack of Orm1/2 phosphorylation by the absence of Swe1 increases inhibition of SPT activity, leading to reduced levels of SLs and rendering *swe1* mutant cells highly sensitive to myriocin.

**FIGURE 6.**
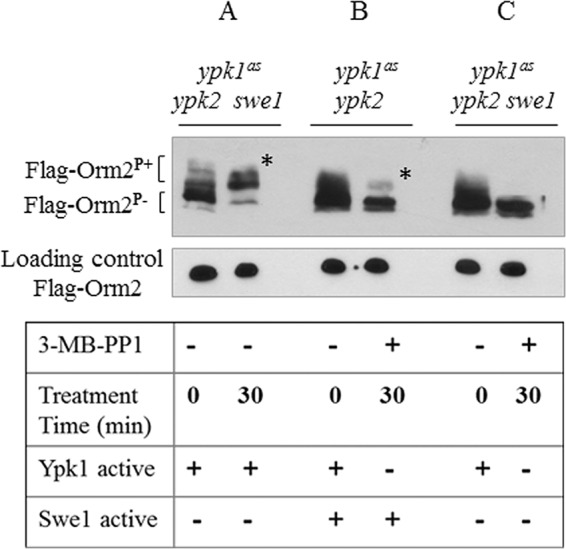
***In vivo* phosphorylation of Orm2 by Swe1.** After separation of total protein extracts from the indicated strains on phosphate affinity gels, Orm2 phosphorylation was detected by immunoblotting. Logarithmically growing cells of *ypk1^as^ ypk2 swe1* and *ypk1^as^ ypk2* mutants expressing 3XFLAG-Orm2 from its chromosomal locus were treated with 670 ng/ml myriocin for 30 min (time point 0 min). The cells were then treated with the Ypk1 inhibitor 3-MB-PP1 for 30 min, as indicated (time point 30 min). *A*, Ypk1 kinase robustly phosphorylates Orm2 in *ypk1^as^ ypk2 swe1* mutant in the absence of the Ypk1^as^ inhibitor 3-MB-PP1. Phosphorylated Orm2 is indicated by an *asterisk. B*, logarithmically growing *ypk1^as^ ypk2* mutant cells were treated with the inhibitor 3-MB-PP1 and myriocin. Phosphorylated Orm2 is indicated by an *asterisk. C*, logarithmically growing *ypk1^as^ ypk2 swe1* mutant cells in the presence of the inhibitor 3-MB-PP1 and myriocin were harvested at the indicated time points. At time point 0, the kinase Ypk1 is still active. After 30 min in the presence of inhibitor, Orm2 phosphorylation in the absence of Ypk1, Swe1, and Ypk2 kinase activities is drastically reduced.

Deletion of both *YPK1* and *YPK2* is lethal (74, 86). Thus, to specifically investigate the contribution of Swe1 to Orm phosphorylation *in vivo*, we used *ypk1^as^ ypk2* and *ypk1^as^ ypk2 swe1* strains, in which Ypk1 can be inactivated by the addition of the inhibitor 3-MB-PP1 (83). Phosphorylated protein species were separated from non-phosphorylated ones on phosphate affinity-tagged SDS gels, which enable better resolution (80), and were detected by immunoblotting; Orm2 phosphorylation was induced *in vivo* by the addition of myriocin (Fig. 6). Consistent with published evidence (50), Orm2 is highly phosphorylated in a strain expressing active Ypk1^as^ upon myriocin-induced inhibition of SPT (Fig. 6*A*). Upon inhibition of Ypk1 activity with 3-MB-PP1 in the *ypk1^as^ ypk2* strain, Orm2 phosphorylation was markedly reduced but not abolished (Fig. 6*B*). Phosphorylation of FLAG-tagged Orm2 was completely absent when, in addition, *SWE1* was deleted in the *ypk1^as^ ypk2* strain upon 30-min treatment with the Ypk1^as^ inhibitor (Fig. 6*C*). Taken together, these data strongly support the notion that the morphogenesis checkpoint kinase Swe1 plays a regulatory role in controlling SL metabolism, either by directly phosphorylating the negative SPT regulator Orm2 or by activating an as yet unidentified kinase other than Ypk1 or Ypk2 that inhibits Orm2. A possible contribution of the kinase Npr1, which is regulated via the nutrient-sensitive TORC1 pathway, on Orm2 phosphorylation under the conditions examined cannot be ruled out; Ypk1- and Npr1-mediated phosphorylation of Orm2 are independent of each other (49). The presence of a doublet FLAG-Orm2 band observed on the Western blot in the absence of the kinases Ypk1 and Swe1 that may be attributed to Npr1 phosphorylation was also observed in reports published earlier (50, 83).

##### Differential Roles of Swe1, Ypk1, and Ypk2 Kinases in Regulating SPT Activity

**FIGURE 7.**
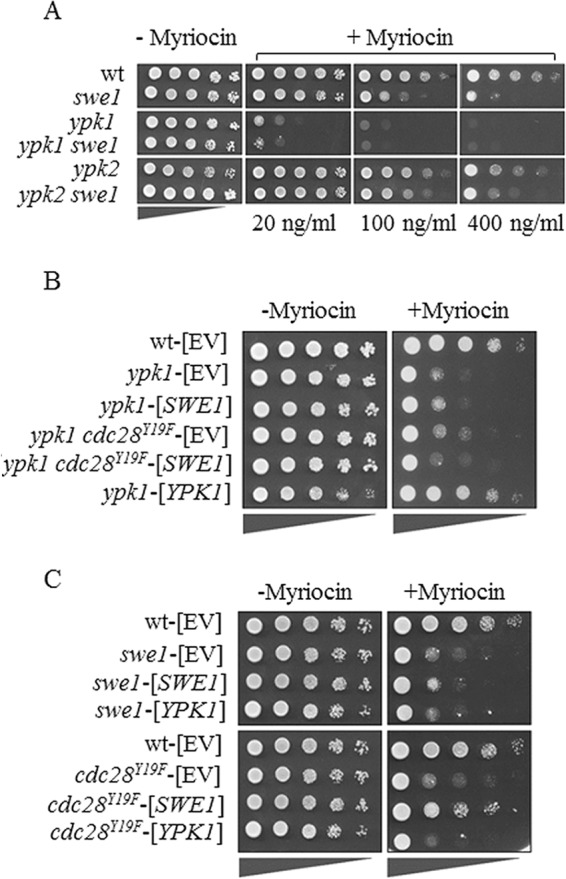
**Swe1 and Ypk1 kinases regulate Orm proteins by independent pathways.** *A*, *swe1*, *ypk1*, and *ypk2* deletion strains display different sensitivities against myriocin. *B*, overexpression of *SWE1* does not rescue growth of *ypk1* and *ypk1 cdc28^Y19F^* mutants in the presence of the SPT inhibitor myriocin. Overexpression of *YPK1* rescues the growth defect of the *ypk1* strain but does not rescue growth of *swe1* and *cdc28^Y19F^* mutants in the presence of myriocin. *C*, overexpression of *SWE1* rescues the myriocin-induced growth defect of both *swe1* (partially) and *cdc28^Y19F^* mutants. *EV*, wild type transformed with pYEX4T-1 empty vector.

The deletion of either Swe1, Ypk1, or Ypk2 (paralog of Ypk1) renders yeast cells sensitive to SPT inhibition; however, the sublethal doses for each mutant differ significantly (Fig. 7). The *ypk1* mutant was most sensitive and showed a growth defect at concentrations of 20–50 ng/ml myriocin, which did not affect growth of the *swe1* or *ypk2* mutants. For the *swe1* mutant, the sublethal dose of myriocin was determined to range from 100 to 200 ng/ml, and the *ypk2* mutant showed a growth defect only at concentrations higher than 400 ng/ml. Importantly, the double deletion mutants *ypk1 swe1* and *ypk2 swe1* were more sensitive to myriocin than the single deletion mutants, indicating synergistic effects (Fig. 7*A*). We suspect that the different sensitivities toward SPT inhibition are due to the different relative impact of each kinase in regulating Orm phosphorylation and, therefore, SPT activity. Ypk1 kinase, as reported (50), appears to be the most active kinase for Orm-SPT regulation, followed by Swe1 and presumably by Ypk2, which is a Ypk1 paralog and likely to share a similar function (74, 86).

Because Swe1 affected Orm2 phosphorylation *in vivo*, we investigated whether the Swe1-Orm and the Ypk1-Orm pathways for the regulation of SL synthesis were connected. Growth tests in the presence of myriocin (Fig. 7*A*) already indicated additive effects of inhibition, suggesting independent pathways. To test whether Swe1 kinase could compensate for the deletion of *YPK1*, *SWE1* was overexpressed in *ypk1* and *ypk1 cdc28^Y19F^* mutant strains. As shown in Fig. 7*B*, the lack of viability in the presence of myriocin was rescued neither by *SWE1* overexpression in the *ypk1* nor by *SWE1* overexpression in the *ypk1 cdc28^Y19F^* mutant. The data suggest that Swe1 cannot compensate for the lack of Ypk1 in Orm phosphorylation (Fig. 7*B*). Similarly, overexpression of *YPK1* did not suppress the myriocin sensitivity of the *swe1* or *cdc28^Y19F^* mutants (Fig. 7*C*). In contrast, overexpression of *SWE1* rescued the myriocin-induced growth defect in both *swe1* (partially) and *cdc28^Y19F^* mutant strains. These data clearly indicate that the Swe1 and Ypk1 kinases regulate SPT activity by different pathways and cannot compensate, when overexpressed, for each other's absence. In addition, the myriocin sensitivity of *cdc28^Y19F^* mutants, which do not respond to inactivation by Swe1, indicates the requirement for a critical level of SLs for normal cell growth if the Swe1/Cdc28 checkpoint remains unchecked.

## Discussion

Multiple functional connections between sphingolipids and the cell cycle exist; however, the specific mechanism coordinating sphingolipid homeostasis and cell cycle progression is not clear (23). The first evidence for an involvement of the morphogenesis checkpoint kinase Swe1 in regulating sphingolipid metabolism came from studies on the *ISC1*-encoded inositol sphingolipid phospholipase C. The *isc1* deletion mutant is sensitive to genotoxic stress-inducing agents, and growth arrest is mediated by activation of Swe1 (54, 87). Under these conditions, Orm2 protein levels increase, and a feedback loop is activated, leading to Orm1/2 dephosphorylation and, as a consequence, SPT inhibition (88). Furthermore, negative genetic interaction was observed between a conditional allele of *LCB1* and deletion of *SWE1* (52, 53).

Our study shows that absence of the Swe1-mediated cell cycle checkpoint results in decreased levels of SLs and increased sensitivity against the SPT inhibitor myriocin. A similar sensitivity to myriocin of the *cdc28^Y19F^* mutant underscores the intricate autoregulatory loop that controls Swe1 activity by Cdc28. These results suggest that in growing cells, (i) a homeostatic level of *de novo* synthesized SLs has to be attained to proceed through the cell cycle, and (ii) one of the functions of the Swe1 kinase checkpoint is to ensure that an adequate amount of SLs is generated by the cells. Because Swe1 regulates a broad range of reactions, we confirmed that the increased myriocin sensitivity of Swe1-deficient mutants was indeed due to a lack of LCB synthesis; the addition of PHS, which bypasses the SPT reaction, fully restored growth of *swe1* or *cdc28^Y19F^* mutants in the presence of the drug.

Absence of the Swe1 kinase diminishes SPT activity in a similar manner as the kinase Ypk1 described previously (50), suggesting that the key regulators of SPT activity, the Orm proteins, may be targets of Swe1 phosphorylation. This was indeed found to be the case, because deletion of *ORM1* and *ORM2* suppresses the myriocin sensitivity of the *swe1* mutant strain. Notably, overexpression of *SWE1* only partially rescued the myriocin-induced growth defect of the *swe1* deletion mutant due to its inhibitory phosphorylation of Cdc28, which is known to induce a G_2_/M arrest, an elongated cell phenotype, and decreased vegetative growth (58, 82). However, full rescue of the myriocin sensitivity by *SWE1* overexpression was achieved in the *cdc28^Y19F^* mutant background in which the checkpoint function of Swe1 is uncoupled from its regulatory impact on SPT activity. This observation further supports the notion of a positive regulatory role of the kinase in regulating sphingolipid homeostasis.

Swe1, either directly or indirectly, promotes phosphorylation of Orm2, thus mitigating the inhibition of SPT, similar to Ypk1 (Ypk2) (50); however, Swe1 and Ypk1/Ypk2 kinases function in independent pathways because neither enzyme was able to complement for the absence of the other when overexpressed. The myriocin sensitivities of the kinase mutants were also found to be different, which reflects apparent differences in their quantitative impact on Orm phosphorylation and SPT regulation. Orm1/2 are known to be phosphorylated at multiple sites (42), and each kinase may contribute to a particular phosphorylation state in response to cellular demands. A large scale analysis of protein phosphorylation in yeast indicated that both Orm1 and Orm2 are direct phosphorylation targets of the Swe1 kinase (51). However, attempts to reconstitute Orm2 phosphorylation by Swe1 *in vitro* were thus far unsuccessful, leaving open the question as to whether its effect *in vivo* is direct. Orm2 phosphorylation is directly influenced by the levels of PHS, and exogenous supplementation of PHS stimulates Orm2 dephosphorylation, indicating that sphingoid bases are the active compounds modulating SPT activity in a feedback loop (83). Total levels of LCBs and specifically C20-PHS were significantly lower in the *swe1* mutant strain as compared with the wild type. The differences between *ypk1* and *swe1* phenotypes with respect to sphingolipid intermediates are intriguing; if Swe1 is acting directly on the Orm proteins and thereby derepressing SPT, it is currently unclear why the chain length distribution of the LCBs actually differs. This finding is subject to ongoing studies. We suggest that lipid-dependent conditions that are unfavorable for cell cycle progression activate the Swe1 kinase-mediated checkpoint. Upon their phosphorylation, Orm inhibition of SPT is alleviated, and the *de novo* synthesis of SLs increases. When SL homeostasis conducive for cell growth is achieved, the Orm proteins are dephosphorylated, and the Swe1 kinase checkpoint is inactivated, allowing the cells to proceed through the cell division cycle.

Most notably, Swe1 activity itself is regulated by SLs that are derived from precursors generated by the breakdown of triacylglycerols by the lipases, Tgl3 and Tgl4 (76, 89). The absence of lipolysis leads to activation of Swe1, which in turn phosphorylates and inactivates Cdc28 at the onset of bud formation in cells entering the cell cycle from G_0_ (75). This places Swe1 function in the center of cell cycle regulation by sphingolipids and suggests that it is the missing link in regulating the sphingolipid rheostat in growing cells.

## Author Contributions

S. D. K. and T. D. conceived and coordinated the study and wrote the paper. N. C. designed, performed, and analyzed experiments shown in Figs. 2–7 and wrote the paper. G. H. designed/constructed strains/plasmids and provided technical assistance for experiments shown in Figs. 2 and 5. N. S. designed/constructed strains/plasmids and provided technical assistance for experiments shown in Fig. 6. K. G. performed/analyzed experiments shown in Fig. 4. All authors reviewed the results and approved the final version of the manuscript.

## Footnotes

4 The abbreviations used are:

SL: sphingolipid
PHS: phytosphingosine
SPT: serine palmitoyltransferase
TOR: target of rapamycin
LCB: long-chain base
3MB-PP1: 1-(1,1-dimethylethyl)-3-[(3-methylphenyl)methyl]-1*H*-pyrazolo[3,4-d]pyrimidin-4-amine.
